# Microvesicular Caspase-1 Mediates Lymphocyte Apoptosis in Sepsis

**DOI:** 10.1371/journal.pone.0090968

**Published:** 2014-03-18

**Authors:** Matthew C. Exline, Steven Justiniano, Jennifer L. Hollyfield, Freweine Berhe, Beth Y. Besecker, Srabani Das, Mark D. Wewers, Anasuya Sarkar

**Affiliations:** 1 Davis Heart and Lung Research Institute, Pulmonary, Allergy, Critical Care and Sleep Medicine Division, Wexner Medical Center, The Ohio State University, Columbus, Ohio, United States of America; IISER-TVM, India

## Abstract

**Objective:**

Immune dysregulation during sepsis is poorly understood, however, lymphocyte apoptosis has been shown to correlate with poor outcomes in septic patients. The inflammasome, a molecular complex which includes caspase-1, is essential to the innate immune response to infection and also important in sepsis induced apoptosis. Our group has recently demonstrated that endotoxin-stimulated monocytes release microvesicles (MVs) containing caspase-1 that are capable of inducing apoptosis. We sought to determine if MVs containing caspase-1 are being released into the blood during human sepsis and induce apoptosis..

**Design:**

Single-center cohort study

**Measurements:**

50 critically ill patients were screened within 24 hours of admission to the intensive care unit and classified as either a septic or a critically ill control. Circulatory MVs were isolated and analyzed for the presence of caspase-1 and the ability to induce lymphocyte apoptosis. Patients remaining in the ICU for 48 hours had repeated measurement of caspase-1 activity on ICU day 3.

**Main Results:**

Septic patients had higher microvesicular caspase-1 activity 0.05 (0.04, 0.07) AFU versus 0.0 AFU (0, 0.02) (p<0.001) on day 1 and this persisted on day 3, 0.12 (0.1, 0.2) versus 0.02 (0, 0.1) (p<0.001). MVs isolated from septic patients on day 1 were able to induce apoptosis in healthy donor lymphocytes compared with critically ill control patients (17.8±9.2% versus 4.3±2.6% apoptotic cells, p<0.001) and depletion of MVs greatly diminished this apoptotic signal. Inhibition of caspase-1 or the disruption of MV integrity abolished the ability to induce apoptosis.

**Conclusion:**

These findings suggest that microvesicular caspase-1 is important in the host response to sepsis, at least in part, via its ability to induce lymphocyte apoptosis. The ability of microvesicles to induce apoptosis requires active caspase-1 and intact microvesicles.

## Introduction

Over 500,000 people develop sepsis annually in United States alone, resulting in 175,000 fatalities [1], [2]. Despite increased awareness and investigation, there are few specific therapies for sepsis and the putative mechanisms of sepsis-related morbidity and mortality are poorly understood. Sepsis is associated with immune dysregulation, initially producing numerous pro-inflammatory mediators which can result in multi-organ dysfunction syndrome (MODS) [3] and later a hypoimmune phase characterized by immune cell apoptosis, especially of lymphocytes [4]. Previous investigations have demonstrated that pharmacologic blockade of caspase-1 improves organ function, reduces lymphocyte apoptosis and increases survival in animal models of sepsis [5], [6]. Our own work specifically showed that pharmacologic blockade of caspase-1 activity reduced splenocyte cell death in murine sepsis [5]. The precise role of caspase-1 in human sepsis remains to be elucidated.

Caspase-1 was first described as IL-1 converting enzyme (ICE), the enzyme responsible for processing and activating proIL-1β to its mature form [7]–[9]. However, its structural homology to *C. elegans* death genes prompted the discovery of a class of proteases termed caspases, which function in inflammation and apoptotic cell death [10]–[12]. In this context, caspase-1 has been associated with both inflammation and apoptosis in sepsis [5], [13]–[15].

Caspase-1 regulation depends upon the assembly of a protein complex termed the inflammasome [16], [17]. The inflammasome is a multi-protein platform that assembles in response to either pathogen-associated molecular patterns (PAMPs) or intrinsic host factors such as uric acid [18]–[20]. At its core, the inflammasome consists of caspase-1, an adaptor protein, apoptosis-associated speck-like protein containing a CARD (ASC), and a member of the NOD-like receptor (NLR) family of pathogen sensing proteins [17], [21], [22]. Activation of the inflammasome results in cleavage of caspase-1 from its precursor form to the active form and subsequent processing of IL-1β and IL-18 [23]–[25]. The activation of caspase-1 has been linked to infection-induced cell death and apoptosis [15], [26]–[31]. Furthermore, caspase-1 deletion or functional inhibition has been linked to survival in animal models of endotoxin shock [5], [32]. However, the mechanism by which caspase-1 induces lymphocyte apoptosis in the context of sepsis remains poorly understood.

Importantly, we have recently documented that caspase-1 can be released from mononuclear phagocytes in a microvesicular encapsulated form [31]. For example, MVs from endotoxin treated monocytes can induce smooth muscle cell death in tissue culture models. This effect requires active caspase-1 presentation by intact MVs. The present study was designed to expand upon this observation by analyzing the role of microvesicular caspase-1 in an *ex vivo* study of plasma microparticles released during human sepsis. We hypothesized that MVs may serve to package and deliver active caspase-1 and other inflammasome components during human sepsis thereby contributing to the lymphocyte apoptosis characteristic of sepsis. To test this hypothesis, we examined septic patients for the presence of MVs containing caspase-1 and the ability of these MVs to induce cell death in distal tissues. In addition, we used an *ex-vivo* model of sepsis to better characterize the ability of these caspase-1 containing MVs to induce apoptosis.

## Materials and Methods

### Reagents

Bacterial lipopolysaccharide (LPS) was obtained from Alexis (Detroit, MI). RPMI 1640 and phosphate buffered saline (PBS) were purchased from Cellgro, Mediatech, Inc (Manassas, VA), and fetal bovine serum (FBS) was obtained from Atlas Biological (Fort Collins, CO). The caspase-1 inhibitor, YVAD-cmk was purchased from Enzyme Systems, (Irvine, CA). All other reagents were obtained from Sigma-Aldrich (St. Louis, MO) unless otherwise specified.

### Microvesicle isolation and identification

MVs were isolated from either conditioned medium or plasma using ultracentrifugation. Conditioned medium or plasma was first centrifuged at 1000 g for 5 min and then at 15,000 g for 15 min to remove cells, cell debris and aggregates. The subsequent supernatant was then centrifuged at 100,000 g for 1 h. Pelleted vesicles were then washed by resuspending them in PBS and again spinning at 100,000 g for 1 h [31]. MVs were then subjected to flow cytometry. Briefly, MVs were sized by comparison to calibrations of the flow cytometer using beads ranging from <0.1–1 micron from Spherotech Inc, IL using the manufacture's protocol. The flow cytometry data had previously been validated by transmission electron microscopy (TEM) as described in our previous work [26]. Pelleted MVs were then subjected directly to western blotting, caspase-1 enzymatic assay or added to fresh lymphocytes.

### ELISA

Total caspase-1 was detected using Quantikine Caspase-1 Immunoassay DCA100 (R&D Systems, Minneapolis, MN). Goat anti-rabbit IgG (H+L)-HRP Conjugate (Bio-Rad, Hercules, CA) and TMB Microwell Peroxidase Substrate System (Kirkegaard and Perry, Gaithersburg, MD) was used for quantification.

### Immunoblots

Microvesicles isolated from *ex vivo* stimulated whole blood were analyzed for the presence of caspase-1 by immunoblots. Quantification of protein in the MVs was performed using densitometry with Quantity ONE. Densitometric analyses of caspase-1 were referenced to known caspase-1 concentration of 75 µg of THP1 cell lysate.

### Caspase-1 activity

For caspase-1 activity assay, monocytes were isolated from buffy coats and cultured at a concentration of 10^7^ cells/ml. Cells were then stimulated with LPS (1 µg/ml) and supernatants were collected from each well. Cells were spun at 1000 g at 4°C for 10 min to collect the supernatant. Supernatants were then subjected to caspase-1 enzymatic assay [33], [34]. 50 µl of sample were mixed with 50 µl of an assay buffer (50 mM HEPES (pH 7.4), 100 mM NaCl, 0.1% 3-[(3-cholamidopropyl)dimethylammonio]-1-propanesulfonate, 20% glycerol, 10 mM DTT and 0.1 mM EDTA) and 5 µl of 1 mM Ac-WEHD-AFC. This mixture was placed in a well of a Costar 96-well flat bottom plate (Corning Glass, Corning, NY) and immediately subjected to kinetic fluorometric assay for 2 h at room temperature using a Cytofluor 4000 fluorometer (Perspective, Framingham, MA) with filters of 360 nm excitation and 460 nm emission. The linear change of the fluorescence of hydrolyzed free AMC per time and the protein concentrations of the assayed samples were used for calculating caspase activity.

### Demographics for Septic Patients and Critically Ill Non-septic Patients

Patients admitted to the intensive care unit (ICU) were screened for eligibility. Inclusion criteria included: age ≥18 years, need for mechanical ventilation, and all patients were enrolled within 24-hours of onset of mechanical ventilation and in the case of septic patients within 24-hours of sepsis recognition. Generally, patients were SIRS positive at presentation and sepsis onset was determined as the time from recognition or suspicion of infection by treating team documented either in written progress notes or by the order for initiation of antibiotics. Patients were assigned to groups based on blinded, retrospective review by two critical care physicians (MCE and BYB). The purpose of retrospective review was to correctly assign patients that were started on antibiotics at time of enrollment, but rapidly had antibiotics stopped when treating team effectively excluded infection or patients that were not on antibiotics at time of enrollment, but had a subsequent infections etiology discovered. Those that met consensus criteria for sepsis [35] (2/4 SIRS criteria and suspected infection) were eligible for the septic cohort. Patients without known or suspected infection were eligible for control cohort. Control patients could be positive for SIRS criteria as long as there was no evidence of infection. Using a protocol approved by The Ohio State University Biomedical Institutional Review Board (IRB), patients or their surrogate decision-maker were informed of study's risks and benefits and provided written consent to have blood drawn within 24 hours of ICU admission and for those remaining in the ICU a repeat blood draw 48 hours later on study day 3. All consent documentation was retained by the clinical trials office. Exclusion criteria included: prisoner status, onset of sepsis more than 24 hours prior to admission, pregnancy, and declined participation. Patients' severity of illness was calculated using the SAPS II scoring system. Survival is defined as survival to hospital discharge.

### Statistical analysis

A cohort of 50 patients was chosen *a priori* based on our previous work [36] and powered to detect a difference between inflammasome constituents in sepsis versus critically ill controls. Data are represented as the median (interquartile range) for at least three independent experiments or mean ± standard deviation. Significance determined by Student t-test if normal distribution or Wilcoxon Rank Sum Test if normality assumption was violated. Logistic regression was performed to determine if severity illness significantly altered associations based on unmatched distribution of the severity of illness in our cohorts.

## Results

### Active Caspase-1 is released in microvesicles during sepsis

**Figure 1:**
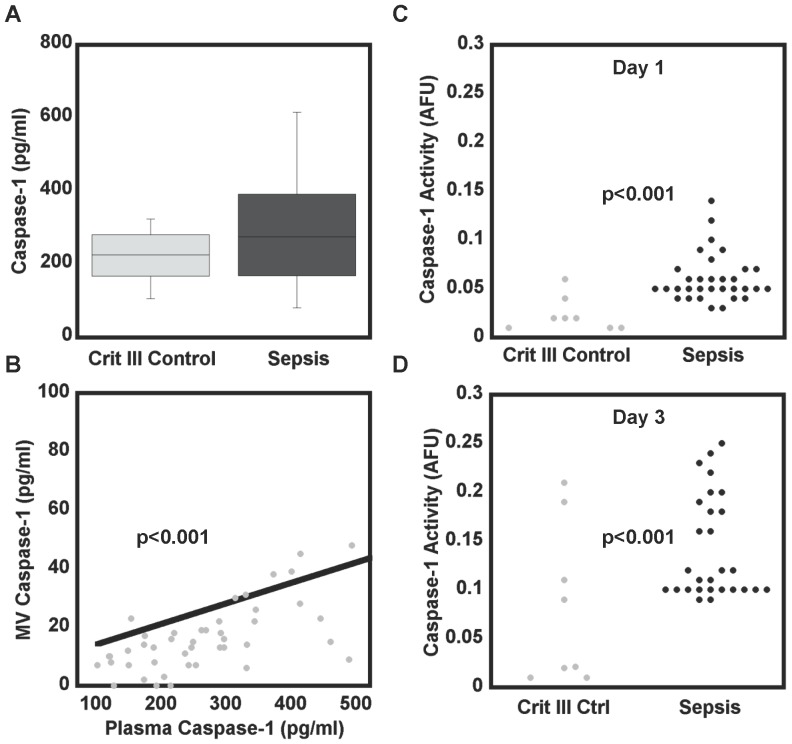
Active caspase-1 is released in plasma microvesicles during sepsis. Plasma samples caspase-1 concentrations between critically ill control patients (n = 16) and septic patients (n = 34) (**A**). Microvesicles isolated on day 1 show a good correlation between plasma caspase-1 and MV caspase-1 (p<0.001) (**B**) There was no significant difference between caspase-1 concentrations in plasma and MVs between control patients and septic patients. However, when analyzed for caspase-1 activity there was significantly higher caspase-1 activity on day 1 (**C**) and on day 3 (**D**) between the septic patients and those that were critically ill (p<0.001 for both days).

To analyze the function of caspase-1, a cohort of patients admitted to the ICU on mechanical ventilation was prospectively recruited. Fifty patients underwent day 1 plasma collection (34 septic, 16 critically-ill non-infected controls) (**Table I**). The control patient admission diagnosis included: seizure (2), stroke (1), lung cancer (1), drug overdose (1), carbon monoxide poisoning (1), pulmonary embolism (1), COPD without pneumonia (3), cirrhosis (1), and cardiovascular disease (5). Caspase-1 was detectable in the plasma of all patients (**Figure 1A**). Isolation of MVs from patient plasma revealed detectable caspase-1 in the microvesicular fraction and there was good correlation between caspase-1 in the plasma and the MVs (R^2^ = 0.88, p<0.001) (**Figure 1B**). There was no significant association between the diagnosis of sepsis and the plasma or MV caspase-1 concentrations. However, caspase-1 is produced as a pro-enzyme that requires cleavage to the active form prior to becoming functional. The caspase-1 concentrations as detected by ELISA represent both active and inactive caspase-1. To determine the presence of active caspase-1, MVs were subjected to a caspase-1 enzymatic assay. There was a strongly significant difference between the caspase-1 activity of the septic patients 0.05 AFU (0.04, 0.07) and the critically ill control patients 0 AFU (0, 0.02) (p = <0.001) with the majority of septic patients showing active caspase-1 in their MVs and the majority of critically ill control patients showing no detectable caspase-1 activity (**Figure 1C**). Not surprisingly due to the lack of association between total concentration and sepsis, there was no correlation between total MV caspase-1 concentrations and caspase-1 activity (data not shown). Looking at the patients that remained in ICU for 48 hours, day 3 MV caspase-1 activity likewise showed elevated levels in septic patients relative to critically ill controls, 0.12 (0.1,0.2) versus 0.02 (0, 0.1) (p<0.001) (**Figure 1D**).

**Table 1:** Demographics Patient Cohort.

	Control	Septic	p-value
Patients	16	34	
Age (yrs.)	59 (50, 74)	55 (46, 71)	ns
Male Sex (%)	9 (56%)	21 (62%)	ns
Shock (%)	6 (37%)	27 (79%)	<0.01
Acute Renal Failure (%)	4 (25%)	15 (44%)	ns
Lactate (mmol/L)	1.7 (1.4, 2.8)	2.2 (1.0, 3.3)	ns
PaO2/FiO2	110 (78, 218)	100 (63, 155)	ns
SAPS II	54 (40, 70)	74 (57, 81)	0.03
Survival*	15 (94%)	21 (62%)	0.02

All values presented as n (%) or median (IQ range) as appropriate.

Demographics of patients enrolled within 24 hours of initiation of mechanical ventilation and/or sepsis onset. Groups were compared by Wilcoxon Rank Sum for continuous variables or Pearson X^2^ for dichotomous variables. Septic patients had a higher incidence of shock and subsequently higher SAPS II scores on the first hospital day. Control patient diagnosis included: seizure (2), stroke (1), lung cancer (1), drug overdose (1), carbon monoxide poisoning (1), pulmonary embolism (1), COPD without pneumonia (3), cirrhosis (1), and cardiovascular disease (5).

Review of our patient demographics reveals a statistically significant difference in the severity of illness between our septic cohort and our critically ill control patients owing to an increase in the incidence of shock in the septic cohort. To account for this, we modeled the predictive ability of the MV caspase-1 activity for determining sepsis with and without inclusion of the severity of illness score. The significance of microvesicular caspase-1 activity persisted adjusting for severity of illness with the change in the point estimate of <10% indicating that the elevated caspase-1 activity seen was specific to sepsis and not due to the overall severity of illness of a patient.

### Septic Patient Microvesicles Induce Lymphocyte Apoptosis

**Figure 2:**
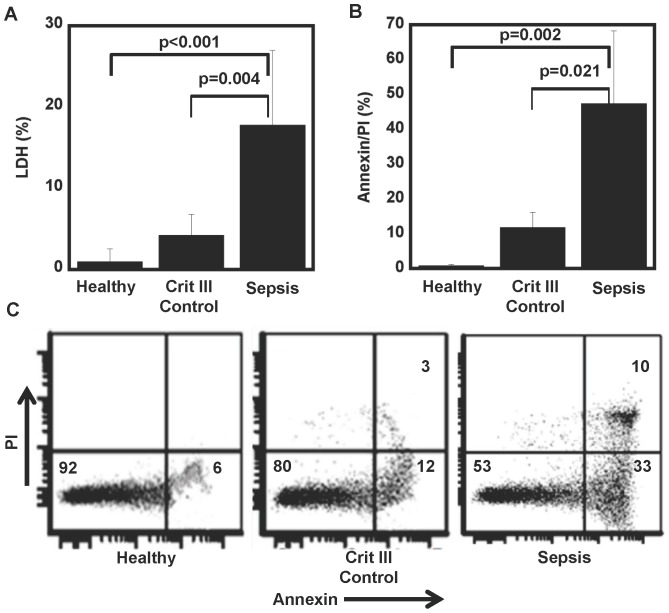
Septic patient microvesicles induce lymphocyte cell death. Plasma was collected from healthy donors (n = 10), critically ill non-septic patients (n = 6), and septic patients (n = 5) and microvesicles were harvested as previously described. Lymphocytes from healthy donors were then incubated overnight with MVs from these respective donors. Cell death was measured by LDH (**A**) and Annexin V/PI assays (**B**). Representative Annexin V/PI assay using flow cytometry exemplified in (**C**). Wilcoxon rank sum test between three groups was significant (p<0.01) for both LDH and Annexin assays and subsequent between group pair-wise comparison via Wilcoxon test are shown. There was no significant difference in cell death or apoptosis between the healthy controls or critically ill control patients, however septic patients had significantly more cell death and apoptosis compared to both healthy controls and critically ill control patients.

To assess the effect that plasma microvesicular inflammasome constituent caspase-1 might have on lymphocyte cell death, a subset of the patients described above (n = 11, 6 septic patients and 5 critically ill non-infected patients) had MVs isolated from fresh plasma and co-cultured for 12 hours with lymphocytes from healthy donors isolated using positive selection by human CD4 magnetic beads from Miltenyi Biotech (Auburn, CA). MVs isolated from the healthy lymphocyte donor plasma served as controls. There was insignificant cell death (by LDH release) at 12 h in the control healthy group 1.0±1.6% and critically ill group 4.3±2.6% (p = n/s) compared with significant cell death in the lymphocytes exposed to septic patient MVs 17.8±9.2% (p<0.001) (**Figure 2A**). Depleting MVs from septic patient plasma greatly reduced the lymphocyte cell death to 7.2±3.3% indicating that the MVs are responsible for the cell death signal. To corroborate the LDH measurements of lymphocyte cell death, flow cytometry using Annexin/PI was also performed. MVs from septic patient plasma induced 47±21% total cell death as compared to 12±4.3% from critically ill patients and 0.9±0.2% from healthy donors (p = 0.018) (**Figure 2B and C**). That this lymphocyte apoptosis was caspase-1 mediated was confirmed by inhibition of plasma caspase-1 activity and abrogation of lymphocyte apoptosis. Inhibition/blocking of active caspase-1 in microvesicles from septic patients reduce healthy donor lymphocyte apoptosis as measured by Annexin/PI by 30% (17.9±6 septic plasma MV vs. 11+2.7 YVAD-cmk treated septic plasma MV). Plasma microvesicles from healthy donors did not induce any significant lymphocyte apoptosis and was also not affected by YVAD treatment.

**Figure 3:**
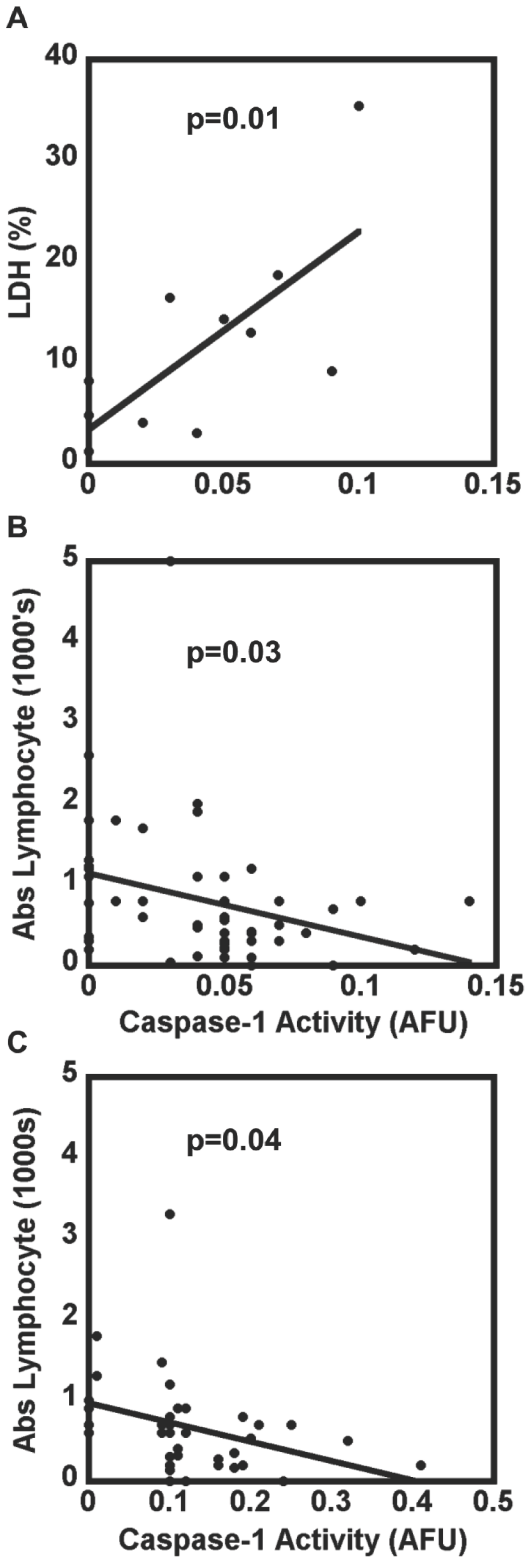
Caspase-1 activity and lymphocyte apoptosis. Caspase-1 activity measured from patient MVs correlated with the ability to induce *ex-vivo* lymphocyte cell death shown in Figure 2 (**A**). Microvesicles isolated from all 50 patients showed a correlation between degree of lymphopenia and microvesicular caspase-1 activity on day 1 (**B**) and day 3 (**C**).

Consistent with these *ex vivo* findings, septic patients from this cohort had significantly lower absolute lymphocyte counts on the day of the microvesicle isolation when compared to our critically ill controls 0.3±0.3 K/ml versus 3.3±4.8 K/ml (p = 0.044). Affirming our findings above, there was a correlation between measured microvesicular caspase-1 activity from the patient and the *ex vivo* lymphocyte cell death induced by the patients' MVs R^2^ = 0.51 (p = 0.014) (**Figure 3A**) suggesting that caspase-1 activity is associated with the sepsis-induced lymphocyte apoptosis. Lastly, in the entire 50 patient cohort there was a significant, albeit weak association between the MV caspase-1 activity and lymphopenia as measured by absolute lymphocyte count on day of enrollment R^2^ = 0.09 (p = 0.03) and day 3 of ICU stay R^2^ = 0.12 (p = 0.04) (**Figure 3B&3C**).

### Microvesicular encapsulation of active caspase-1 essential for its apoptotic property

**Figure 4:**
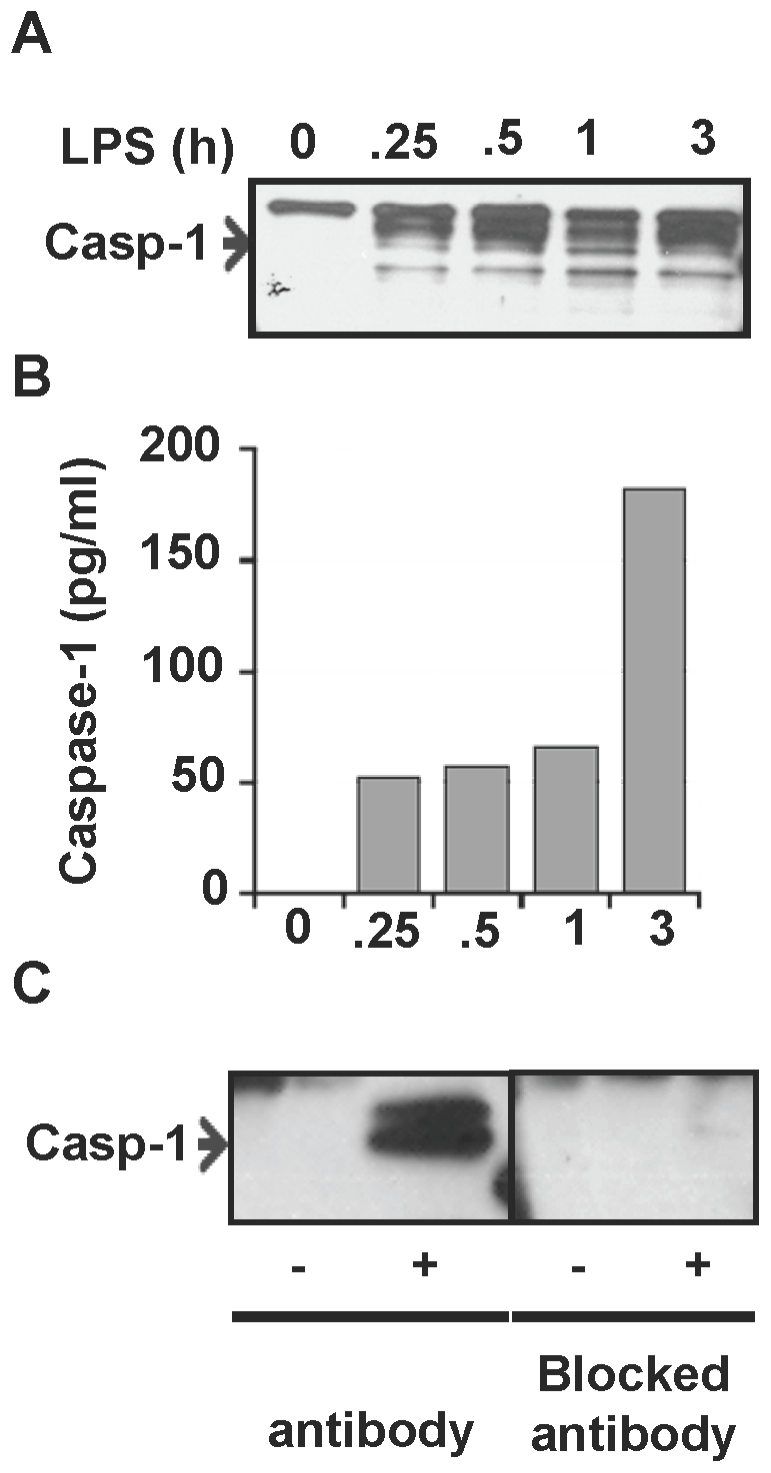
Release of caspase-1 in microvesicles from endotoxin stimulated whole blood. Whole blood was stimulated with LPS for different times (0, 0.25, 0.5, 1 and 3 h) and microvesicles were isolated from the plasma of the stimulated blood. Caspase-1 was detected in the microvesicles by immunoblot (**A**) and ELISA (**B**). To show specificity, caspase-1 detection was also performed after blocking the antisera with excess recombinant caspase-1 before immunoblot (**C**).

Microvesicle shedding is a recognized mode of cytokine release, as described for IL-1β and Fas ligand [37]–[43]. To demonstrate that the apoptosis seen with the lymphocytes co-incubated with septic serum was due to active caspase-1 in MVs, peripheral blood was collected from healthy normal donors using heparin to prevent coagulation. Heparinized whole blood was then either left unstimulated or stimulated with LPS (1 µg/ml) for 0.25, 0.5, 1 and 3 h. This blood was then spun at 2000 rpm for 10 min and plasma was collected. MVs isolated from plasma contained cleaved caspase-1 by immunoblot (**Figure 4A**). The presence of caspase-1 in released vesicles was also confirmed by ELISA (**Figure 4B**). Increased amounts of caspase-1 (1.6–3.3 fold) were detected in MVs from LPS stimulated whole blood as compared to unstimulated blood. The specificity of caspase-1 in MVs from stimulated whole blood was further confirmed by complete inhibition of detection when the antiserum was preincubated with excess recombinant caspase-1 (**Figure 4C**).

**Figure 5:**
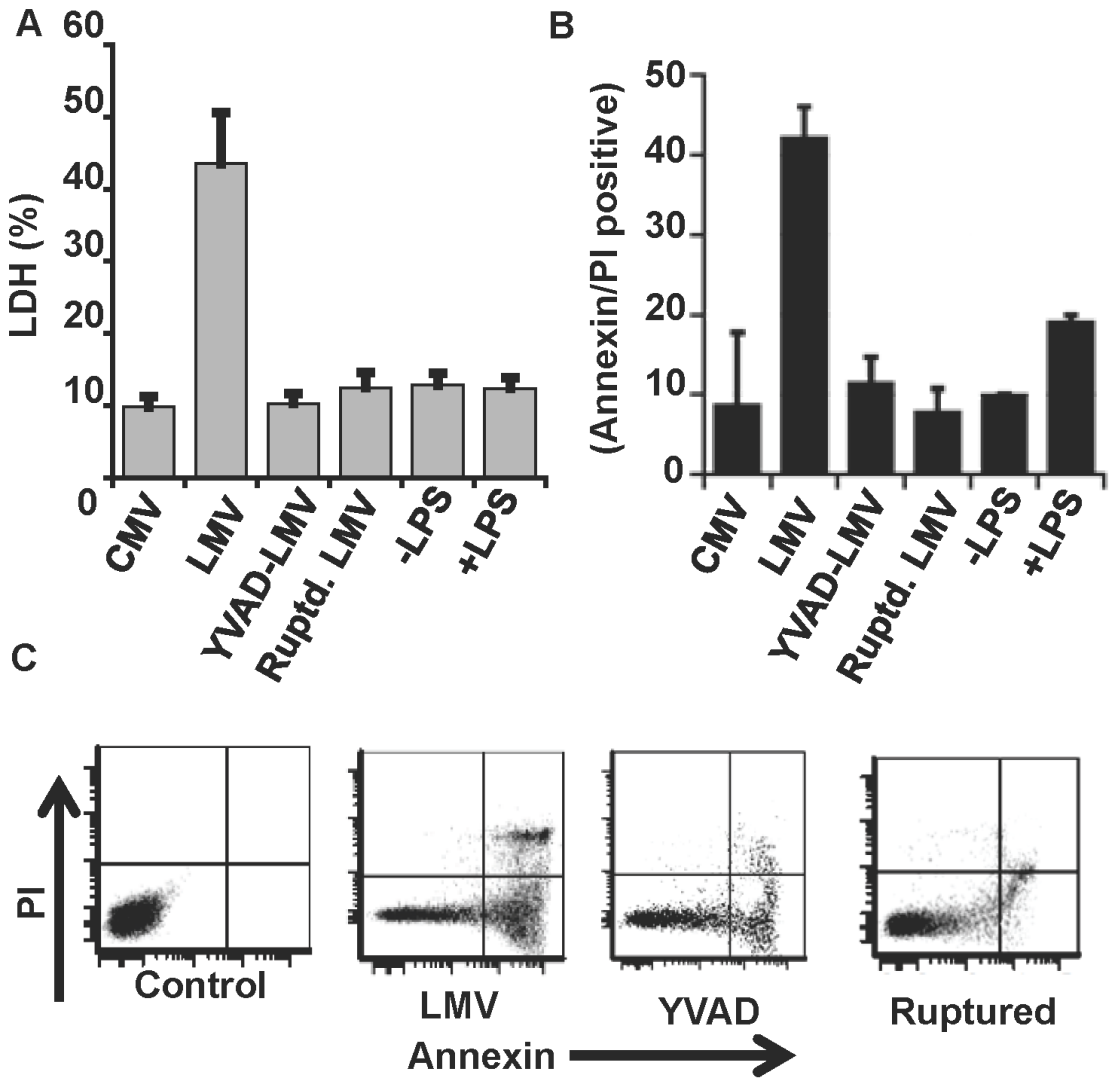
Lymphocyte cell death requires encapsulation of active caspase-1 with intact microvesicles. Whole blood was stimulated with LPS (1 µg/ml) for 1 h and microvesicles were isolated. Lymphocytes from healthy donors were then incubated overnight with microvesicles isolated from unstimulated blood, i.e. control microvesicles (CMV), or LPS (LMV), and LPS + YVAD (YVAD-LMV) treated whole blood. Intact microvesicles from LPS treated whole blood were also disrupted by mild homogenization (Ruptd. LMV) and analyzed for induction of lymphocyte apoptosis. Lymphocytes were also either left unstimulated (-LPS) or subjected to LPS directly and analyzed for cell death. Cell death was measured by LDH (**A**) and Annexin V/PI assays using flow cytometry (**B**) (n = 3). Representative data of apoptosis of lymphocytes by flow cytometry using Annexin V/PI assay (**C**).

To investigate the possibility of lymphocyte cell death induced by caspase-1, MVs isolated from stimulated and unstimulated whole blood (as described above), were then co-cultured overnight with fresh lymphocytes harvested from healthy donors and analyzed for cell death. Compared with unstimulated MVs (CMV), MVs from LPS-stimulated whole blood (LMV) induced lymphocyte cell death as measured by LDH release (43.5±7) (**Figure 5A**). Apoptotic cell death was confirmed by Annexin-V assay (**Figure 5B–C**). Lymphocytes incubated in the presence of MVs isolated from whole blood incubated with LPS and caspase-1 inhibitor, YVAD-cmk, showed significantly less apoptosis than lymphocytes incubated with LPS-stimulated MVs without inhibitor (8±8 vs. 42±3 Annexin/PI positive), suggesting the role of microvesicle encapsulated caspase-1 in induction of lymphocyte apoptosis. Likewise, rupture of the MVs prior to incubation dramatically reduced lymphocyte cell death, suggesting the need for intact MVs for active caspase-1 to target the lymphocyte. Lymphocytes incubated with and without LPS showed apoptotic levels similar to the CMV sample indicating that cell death was due to caspase-1 containing MVs rather than residual contamination of the MVs with LPS.

## Discussion

Sepsis is a disease of both inflammation and cell death. Since caspase-1 has a role in both inflammatory cytokine processing and apoptosis, understanding its regulation is essential to understanding sepsis. The present study confirms our previous findings that in addition to the IL-1β found present by other groups [37], [40], [42], [43], MVs shed from monocytes in endotoxin stimulated whole blood contain inflammasome components, principally caspase-1. Importantly, these inflammasome containing vesicles are capable of inducing apoptotic cell death in healthy donor human lymphocytes. Though caspase-1 is the prime member of the inflammatory caspase family which functions to activate proIL-1β and proIL-18, the current findings reinforce that caspase-1 can also be a significant mediator of apoptotic cell death. This cell death function of caspase-1 has been described for macrophages responding to intracellular pathogens in pyroptosis [26] and as we have shown for splenic B lymphocyte apoptosis in response to sepsis and smooth muscle cell apoptosis in a model of atherosclerosis [5], [31].

This study is, to our knowledge, the first to demonstrate the presence of circulating caspase-1 in the blood of critically ill patients. Though many investigations have shown lymphocyte apoptosis during sepsis, the mechanisms of this apoptotic process are still unclear [13], [44]–[49]. Previous work has demonstrated that serum from patients with septic shock contain circulating factors that can induce cellular apoptosis. However, the source and composition of these factors have yet to be fully elucidated [50]–[53]. Our work implicates caspase-1 as one of the potential apoptotic signaling factors during sepsis.

The fact that caspase-1 circulates in MVs during sepsis is also novel. How sepsis induces a systemic apoptosis of tissue and circulating lymphocytes is unknown. Our finding that the inflammasome is packaged and released in MVs provides a mechanism by which this apoptotic message could be systemically targeted to other cells, though how these MVs find their target tissue still needs to be elucidated. Furthermore, our results demonstrate that MVs isolated from the blood of septic patients can induce apoptosis in healthy lymphocytes and that the degree of apoptotic cell death is directly related to the concentration of active caspase-1 in the microvesicle. Additionally, our *in vitro* work showed that this effect is blocked by inhibition of caspase-1, further implicating caspase-1 as a signal of sepsis-induced apoptosis and confirming previous observations by multiple investigators in animal models [5], [6], [54]–[56].

Microvesicles have been known to be shed by cells during activation or apoptosis and to carry different factors and proteins [31], [37]–[43], [51], [53]. Monocyte/macrophage derived MVs have been reported to transport biologically significant amounts of phosphatidylserine and tissue factor [37], [38], [41]. Members of the caspase-related protease family have been shown to play an important role in apoptosis [5], [31], [57]. However, the specific role of caspase-1 in apoptosis is controversial. Caspase-1 knockout animals are born healthy without detectable morphological abnormalities, whereas caspase-3 deficient animals have major birth defects, particularly neurological defects which imply a role for caspase-3 in developmental apoptosis. Furthermore, we have previously documented that spontaneous monocyte apoptosis is not dependent upon caspase-1 but upon caspase-3 activity [33]. On the other hand, over expression of caspase-1, in a rat fibroblast cell line induces an apoptosis which is blocked by crmA, a cowpox virus protein that inhibits caspase-1 [58]. The involvement of caspase-1 in neuronal cell apoptosis is also well established. Gagliardini *et al*, 1994, observed the ability of a caspase-1 inhibitor to prevent apoptosis induced by nerve growth factor deprivation [59]. Furthermore, caspase-1 has been implicated in the death of *Salmonella* infected dendritic cells and monocyte derived macrophages [15], [60]. Work from our own laboratory has shown the unique role of caspase-1 in regulating sepsis survival by regulating splenic lymphocyte apoptosis [5]. Thus, the present work lends support to the notion that caspase-1 is important in at least selected forms of programmed cell death. Our findings suggest that microvesicular caspase-1 can directly regulate the apoptosis of lymphocytes and that encapsulation of this active caspase-1 in MVs is critical for its function.

There are several limitations to our study that must be mentioned. Overall, this is a small study with only 50 total patients enrolled and due to the prospective nature of our enrollment there was not an even distribution of sepsis and critically ill control patients. We did see a difference in caspase-1 activity in septic patients versus critically ill control patients, however this was not true for all patients examined and there was some heterogeneity in the patient population. This may be due to the fact that sepsis is a clinical syndrome rather than a specific diagnosis. Often, patients admitted to the ICU are started on empiric antibiotics thus meeting criteria for a suspected infection, while it is difficult to confirm the presence of a true infection. This is a limitation of all sepsis research that attempts to use other critically ill patients as a control. Secondarily, though we were able to demonstrate that MVs from septic patient serum induced apoptosis in healthy lymphocytes, we did not demonstrate that the MVs specifically target lymphocytes *in vivo*. Though the correlation between absolute lymphocyte counts and caspase-1 activity suggests this may be true. Our previous work has shown that caspase-1 does not kill native peripheral blood monocytes or macrophages and that lymphocytes are particularly susceptible to sepsis-induced apoptosis compared with other tissues, thus supporting the likelihood of lymphocyte specific targeting.

Our work does not delve into the mechanism by which caspase-1 is killing the lymphocytes. Previous studies from our laboratory have demonstrated that caspase-1 does induce apoptosis during murine models of sepsis and that the apoptotic function is independent of its IL-1β and IL-18 processing function [5]. However, the mechanism by which exogenous caspase-1 induces cell death is poorly understood in general. Does it activate the apoptosome directly or does it act via an intermediary? This will have to be elucidated in future research.

Our analysis of the MVs was incomplete in that we did not evaluate for genetic material such as mRNA which can be encapsulated in MVs nor did we exclude that other cellular death signals were not present in the MVs. Our demonstration that caspase-1 inhibition ameliorated the cell death caused by MVs suggests at the least that caspase-1 is a significant inducer of lymphocyte cell death. We also are unable to characterize whether septic patients had more MVs in circulation or if their MV concentration was similar to critically ill control patients, but that their MVs had a higher concentration of active caspase-1. This will need to be further evaluated in future studies likely in pre-clinical studies.

The final limitation is that our septic patients were considerably more ill than our critically ill patients. This was entirely due to a higher incidence of shock in the septic patients. We adjusted our model to control for this increased severity of illness, and still found a significant relationship between sepsis and elevated MV encapsulated caspase-1, but there is still the possibility that caspase-1 may play less of a role in less severely ill septic patients.

In light of the failure of anti-inflammatory therapy for the majority of septic patients [61], understanding the phenotype of pro-inflammatory and pro-apoptotic sepsis is crucial to develop new therapeutic avenues. Indeed, there was a great deal of heterogeneity in both our septic and critically ill patients that may represent either patient-specific or pathogen-specific factors that influence caspase-1 release. Larger future trials may be needed to further describe these relationships.

In summary, our results demonstrate that caspase-1 plays a central role in the regulation of apoptosis in an *ex vivo* model. This novel apoptotic event is dependent upon encapsulation of exogenous caspase-1 into released MVs that allow targeting of the active enzyme to the cytosol of different cells, in this study lymphocytes. Our work provides an opportunity to further describe a specific mechanism in human sepsis by which apoptosis can be induced. The exact mechanism by which these MVs recognize and target lymphocytes remains to be elucidated.
